# Radiological Findings of Sexual Intercourse Related Emergency Department Admissions: A First Overview

**DOI:** 10.1371/journal.pone.0104170

**Published:** 2014-08-05

**Authors:** Carmen A. Pfortmueller, Adrian C. Schankath, Pasquale Mordasini, Jana Koetter, Roland Wiest, Aristomenis K. Exadaktylos, Stefan Puig

**Affiliations:** 1 Department of General Anaesthesiology, Intensive Care and Pain Management, Medical University of Vienna, Vienna, Austria; 2 University Institute of Diagnostic, Interventional and Pediatric Radiology, University Hospital and University of Bern, Bern, Switzerland; 3 University Institute of Diagnostic and Interventional Neuroradiology, University Hospital and University of Bern, Bern, Switzerland; 4 University Department of Emergency Medicine, University Hospital and University of Bern, Bern, Switzerland; Catholic University of Sacred Heart of Rome, Italy

## Abstract

**Objectives:**

Sexuality is an essential aspect of human function, well-being and quality of life. Many people have sex without complications. However, there are some people who need to seek emergency medical help for related health problems. The aim of this study was to present a first overview of patients who received a radiological examination related to sexual intercourse based emergency department admission.

**Methods:**

Our centralized electronic patient record database was reviewed for patients who had been admitted to our emergency department with an emergency after sexual intercourse between 2000 and 2011. The database was scanned for the standardized key words ‘sexual intercourse’ or ‘coitus’ retrospectively. For all patients identified in the electronic patient record database the radiological examinations were searched for manually in our Radiology Information System, and reviewed by three independent radiologists.

**Results:**

One hundred and twenty nine out of 445 (29,0%) patients received a radiological examination after immediate emergency department admission related to sexual intercourse. Fifty two out of 129 (40.3%) patients had positive radiological findings while 77 (59.7%) did not. Eighty point seven percent (n = 42) of the radiological findings were a sexual intercourse-associated pathology and 19.2% (n = 10) were considered to be incidental findings. Age and male sex positively correlated with radiological imaging workup (p<0.001, respectively p<0.037). The most common sexual intercourse-associated pathology was headache attributed to cerebrovascular insult (n = 21, 40.3%) followed by epididymitis (n = 7, 16.6%) and obstructive uropathy (n = 5, 11.6%). Of the patients with headache attributed to non-traumatic intracranial hemorrhage, subarachnoid hemorrhage (n = 14, 66.6%) was the most common, followed by intracerebral bleeding (n = 4, 19.0%) and one subdural hemorrhage.

**Conclusions:**

Pathological findings are manifold. Cerebral imaging is the most common type of radiological imaging performed. Further prospective and standardized studies should be performed to better evaluate the significance of radiological imaging in this patient collective with the aim to gain better knowledge on what patients profit from what type of radiological imaging when presenting with a sexual intercourse related emergency.

**Advances in Knowledge:**

The present study provides a first overview on radiological findings of sexual intercourse related emergency department admissions.

## Introduction

Sexuality is defined as the possession of the structural and functional traits of sex. It is related to intimacy and procreation [1], [2]. Sexual activity is mechanically dangerous, potentially infectious and stressful for the cardiovascular system [3] and is associated with an increased metabolic demand [1], [4]. In their experiments in the 1960's, Masters and Johnson found that both sexes exhibit radical changes in hemodynamic, ventilatory and myotonic patterns during sexual intercourse [5]. There are four distinct stages of the human sexual response: excitement (initial state of arousal, characterized by increases in muscular tone, heart rate and blood pressure), plateau phase (full arousal immediately preceding orgasm, further increases in muscular tone, heart rate and blood pressure, increased relative vascular resistance), orgasm (climax, associated with muscle spasms, massive elevation of heart rate (up to 175 beats per minute), blood pressure (up to 220 mg systolic) and respiratory rate (up to 40/minute) and resolution (normalizing of physical function) [5], [6]. Despite health benefits of maintaining an active sex life [7], sexual intercourse is associated with morbidity and mortality [2]. Many people do have sex without complications, however, there are some people who need to seek emergency medical help for related health problems [2], [8]. The incidence of health problems in relation to sexual intercourse is not known as the intimate nature of the problem may not be reported to a physician [2]. Even less is known about emergency department admission related to sexual intercourse [2].

To the best of our knowledge no study has ever described radiological examinations related to sexual intercourse-related emergency department admissions. The aim of this study was to give a first overview of radiological findings in patients with sexual intercourse based emergency department admission.

## Materials and Methods

### Setting

Our emergency department is the only Level I centre in a catchment area serving about 1.8 million people and treats more than 35,000 cases per year. Despite slight variations in clinical practice between the physicians in our emergency department, the practical evaluation of patients generally follows the same pattern. Based on actual recommendations the diagnostic and therapeutic management is at the discretion of the attending emergency physician. A team of emergency radiologists is on call 24 hours every day.

### Data collection and data retrospective analysis

Our retrospective data analysis comprised adult patients (≥16 years) admitted to our emergency department in relation to an emergency immediately after sexual intercourse between 1 January 2000 and 31 December 2011. They were identified using the appropriate search string (‘sexual intercourse’ or ‘coitus’) in the anamnesis field of our computerized patient database (Qualicare Office, Medical Database Software, Qualidoc AG, Bern, Switzerland). Since this medical database allows instantaneous retrieval of past diagnostic reports, discharge summaries, consultations and other relevant medical documents or radiographs, the authors were able to retrospectively analyze the reason for presentation, the diagnostic results, and therapeutic procedures initiated in the emergency department. Presentations were only attributed to sexual intercourse when clearly stated so by the patient and sexual intercourse took place a maximum of 24 hours before the begin of symptoms. The following clinical data were extracted from medical records: reason for presentation, clinical features, diagnosis, and, if performed, type of radiological imaging and radiologic findings. No nursing records were consulted. Demographical data such as gender and age were also assessed. All clinical records were reviewed by a specialist in internal medicine and a specialist in emergency medicine. The reason for presentation and diagnosis was extracted according to diagnosis and anamnesis, no ICD 10 coding was used. The diagnosis was categorized into five disciplines (cardiovascular, neurological, trauma, infectious, various complaints). Each patient was only categorized into one group. For all categorizations each specialist had to agree independently. Data on the clinical presentations of patients presenting with sexual activity- related emergency department admissions has been published previously [3]. Our hospital's policy defines adults as patients of 16 years or more, all patients younger than 16 years are treated at a specialized emergency department for children and are therefore not included in the study. Additionally patients with duplicated (n = 23) and incomplete (n = 213) records (lack of medical history) and those with a sexual activity-related health issue not related to the main reason for emergency department admission (n = 170) were excluded from the analysis. For all patients the radiological image where searched in our Radiology Information System manually. The images were interpreted by three independent radiologists (Adrian Schankath, Pasquale Mordasini, Stefan Puig). All specialists had to agree on image interpretation and differentiation between incidental and pathological finding independently otherwise the case was discussed by an expert panel. The radiologist were blinded to the clinical diagnosis.

### Statistical Analysis

All statistical analyses were performed with the SPSS 20.0 Statistical Analysis program (SPSS Inc; Chicago, IL). The data were summarised using descriptive statistics (means, standard deviations, percentages and N's). The differences between patients with and without radiological imaging or patients with positive and incidental radiological findings were compared between injury types using chi-squared tests for categorical variables, t-tests and ANOVA for continuous variables. All p values were two tailed and at a level of significance of 0.05.

### Ethical consideration

The study was approved by the ethical review board of the “canton” ( = district) of Berne, Switzerland. No individual informed consent was obtained, it was waived by the ethics commetee. Patients records were anonymized prior to analysis.

## Results

A total of 445 ED admissions related to sexual intercourse were eligible for our study. Three hundred and eight (69.0%) were men and 137 (31.0%) women. The median age was 28 years (SD 12.92, range 16–71). Patients' characteristics are displayed at Table 1.

**Table 1:** Patients characteristics (n = 445).

	Overall population	Patients with radiological examination
Number of patients (%)	445 (100%)	129 (28.9%)
Mean age, years (SD)	36.83 (14.73)	34 (14.73)
*Gender, n (%)*		
Male	308 (69.0%)	79 (61.2%)
Female	137 (31.0%)	50 (38.8%)
*Radiological examination type, n (%)*		
Head CT		37 (28.7%)
Urogential Sonography		21 (16.3%)
Scrotal Sonography		19 (14.7%)
Cerebral MRI		18 (14.0%)
Abdominal Sonography		17 (13.2%)
Abdominal CT		7 (5.4%)
Vaginal Sonography		4 (3.1%)
Abdominal X-ray		1 (0.8%)
Thoraco-abdominal CT		1 (0.8%)
Chest CT		1 (0.8%)

Hundred and twenty nine out of 445 patients (28.9%) received a radiological examination. Of these, 61.2% (n = 79) were male and 38.8% (n = 50) female. The median age was 34 (SD14.73, range 16–71). Age and male sex positively correlated with radiological imaging workup (p<0.001, respectively p<0.037). The most frequent cause of presentation of patients with radiological examination was neurological symptoms (atraumatic headaches, stroke-like symptoms) (n = 54; 41.9%), followed by symptoms of urogenital tract infections (n = 50, 38.8%) and various complaints (n = 19, 15.5%). Five cases were related to trauma and one to aortic dissection (see table 2). Having neurological symptoms positively correlated with radiological imaging (p<0.001) whereas trauma negatively correlated with radiological work-up (p<0.008). The most common radiological examination performed was head CT (n = 37, 28.7%) followed by urogenital sonography (n = 21, 16.3%), scrotal sonography (n = 19, 14.7%), cerebral MRI (n = 18, 14.0%), abdominal sonography (n = 17, 13.2%), abdominal CT (n = 7, 5.4%), vaginal sonography (n = 4, 3.1%) and one abdominal x-ray, one thoraco-abdominal CT and one chest-CT. Cranial imaging (CT and MR) where associated with age (p<0.001) but not with gender (p<0.20).

**Table 2:** Overview on the four most common diagnoses in patients who received radiological examinations (n = 129).

cardiovascular (n = 1, 0.8%)	trauma (n = 5, 3.1%)	neurological (n = 54, 41.9%)	infectious (n = 50,38,8%)	various complaints (n = 19, 15.5%)
aortic dissection (1, 100%)	sexual assault (2, 40%)	Post-coital headache (26, 48.1%)	urethritis (14, 28.0%)	non-specific abdominal pain (13, 65.0%)
	penile hematoma (1, 20.0%)%)	atraumatic subarachnoid hemorrhage (14, 25.9%)	epididymitis (10, 20.0%)	non-traumatic, non-infectious scrotal pain (2, 10.0%)
	ruptured ovarian cyst (1, 20.0%)	transient global amnesia (10, 18.5%)	urethritis (10, 20.0%)	sexual toy accident (2, 10.0%)
	hip luxation (1, 20.0%)	ischemic cerebrovascular insult (3, 5.6%)	Pyelonephritis (5, 10%)	sexual assault (1, 5%)

A total of 52 (40.3%) patients had positive radiological findings whereas 77 (59.7%) did not. Of the patients with positive radiological findings 30 were male (57.7%) and 22 (42.3%) female. Their median age was 35.5 (SD 14.61, range 18–67). There was no significant age or sex difference between the patients with and without positive radiological findings (p<0.47, respectively 0.8).

**Figure 1:**
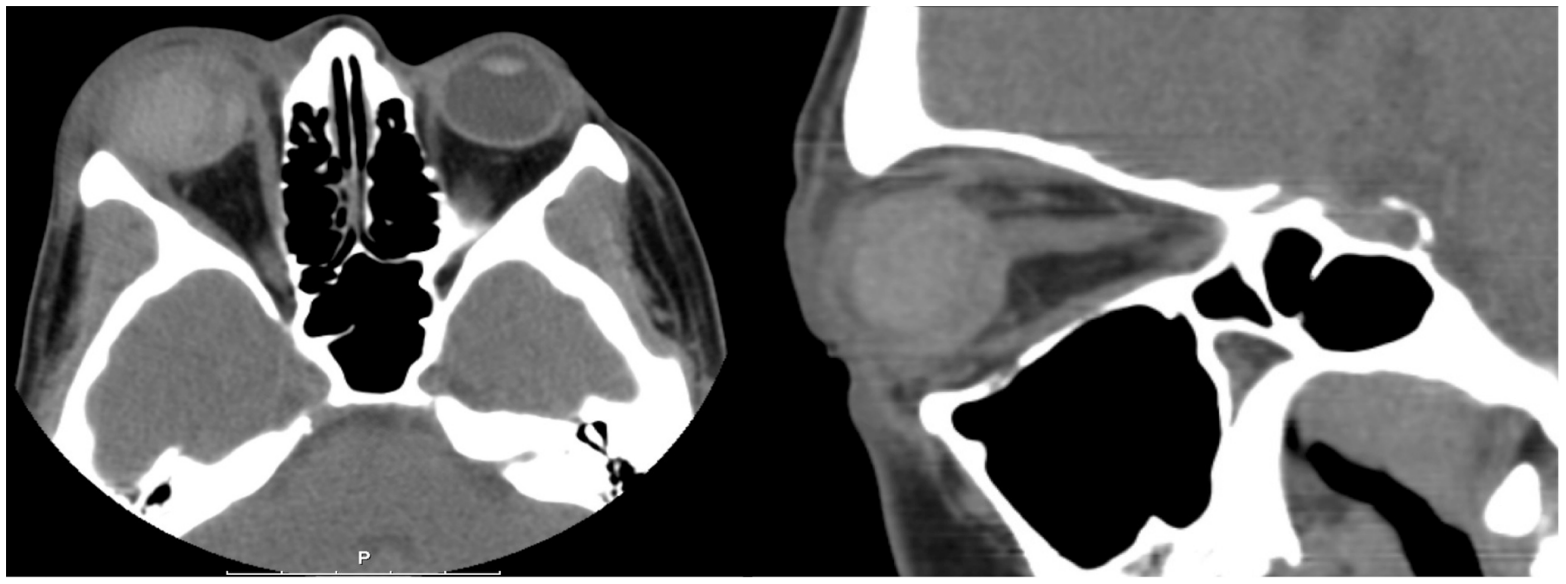
29 year old woman presenting with loss of eye sight and ocular pain. Non-contrast enhanced CT revealed an anterior bulbus perforation with hemorrhage in the anterior chamber and the vitreous humor. No pathological changes retrobulbar and no foreign body was noted.

**Figure 2:**
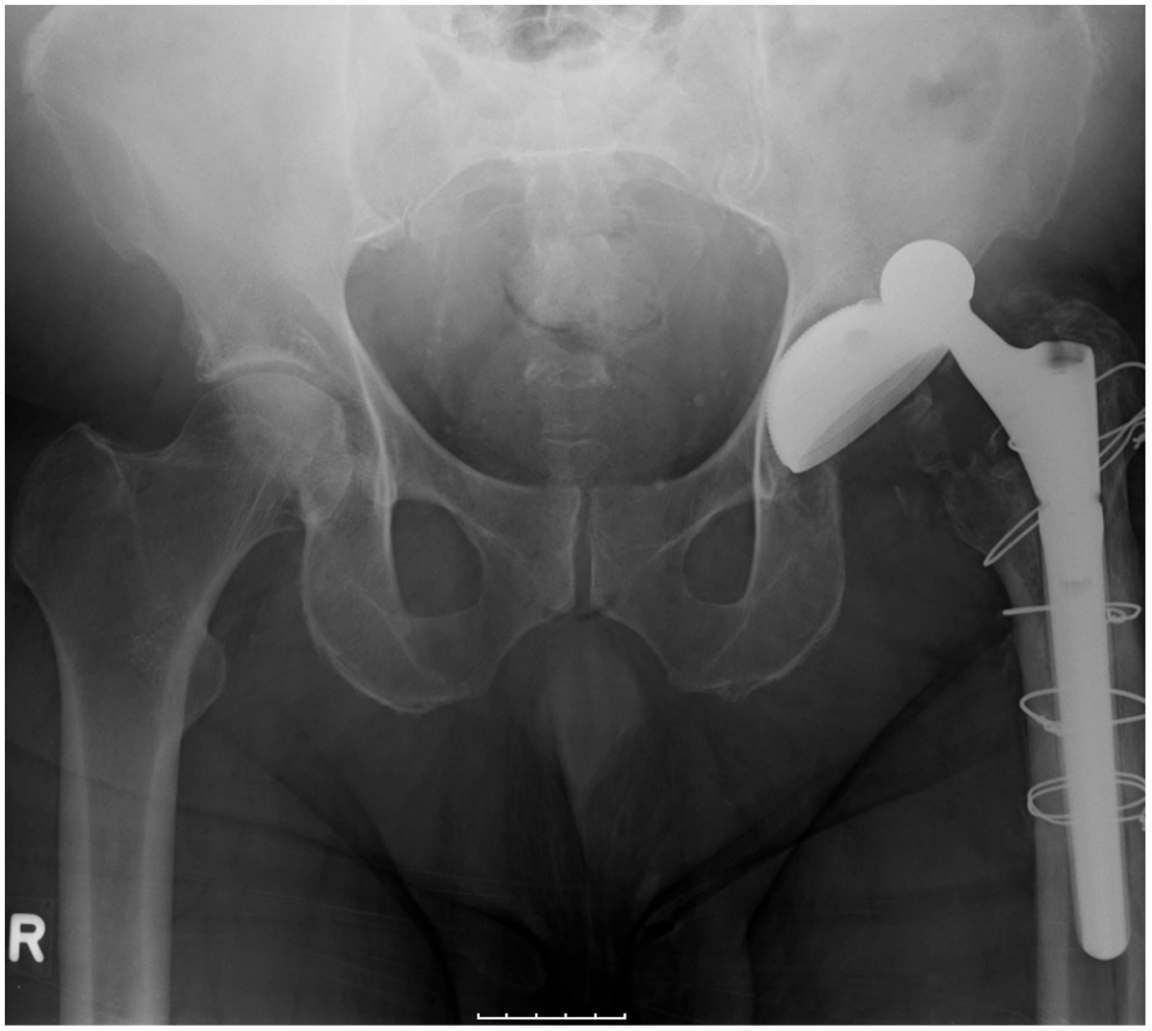
58 year old man presenting with hip pain. Conventional x-ray showed a dislocated total hip replacement on the left.

**Figure 3:**
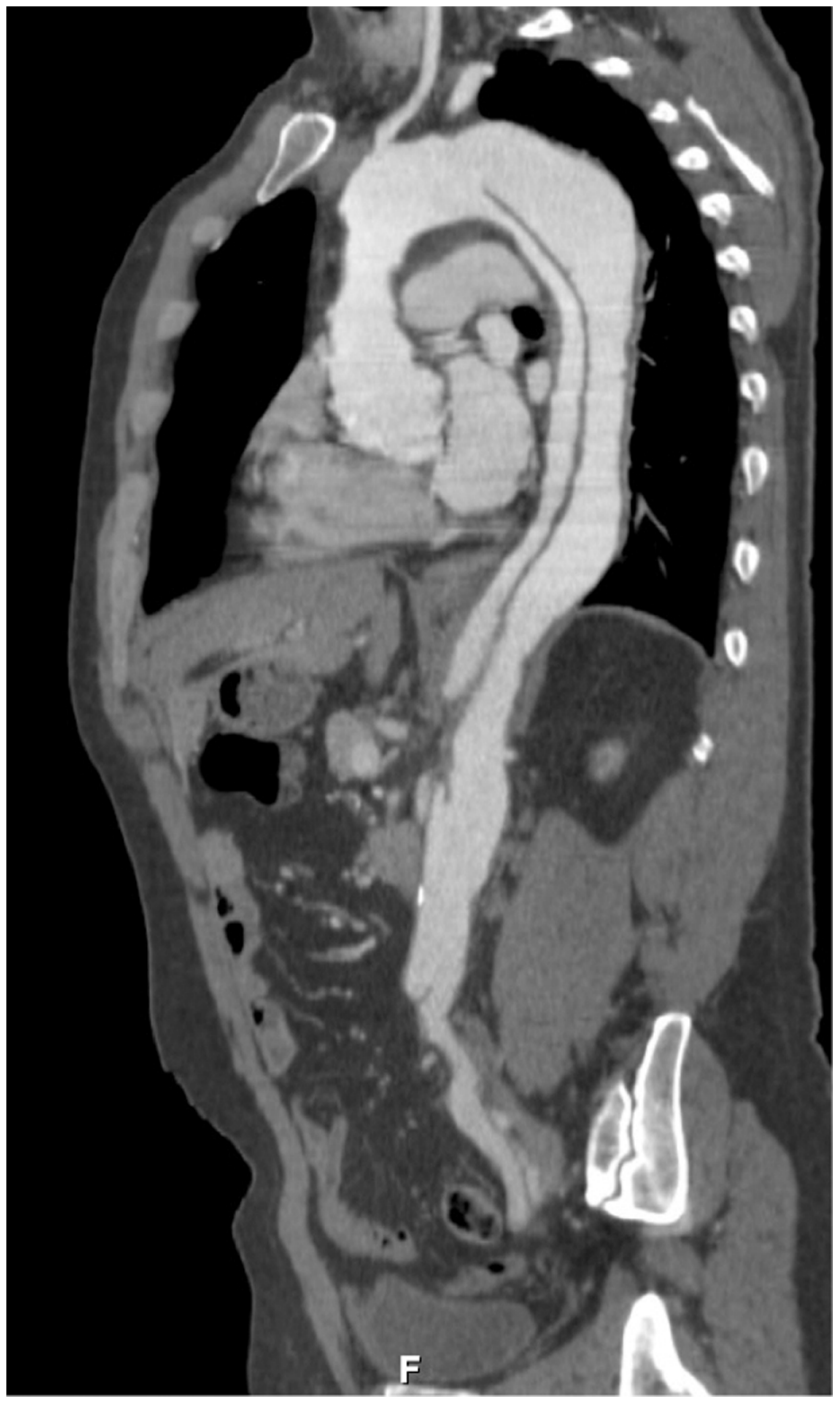
52 year old man presenting with acute chest pain. Contrast enhanced CTA showed a type-b aortic dissection.

**Figure 4:**
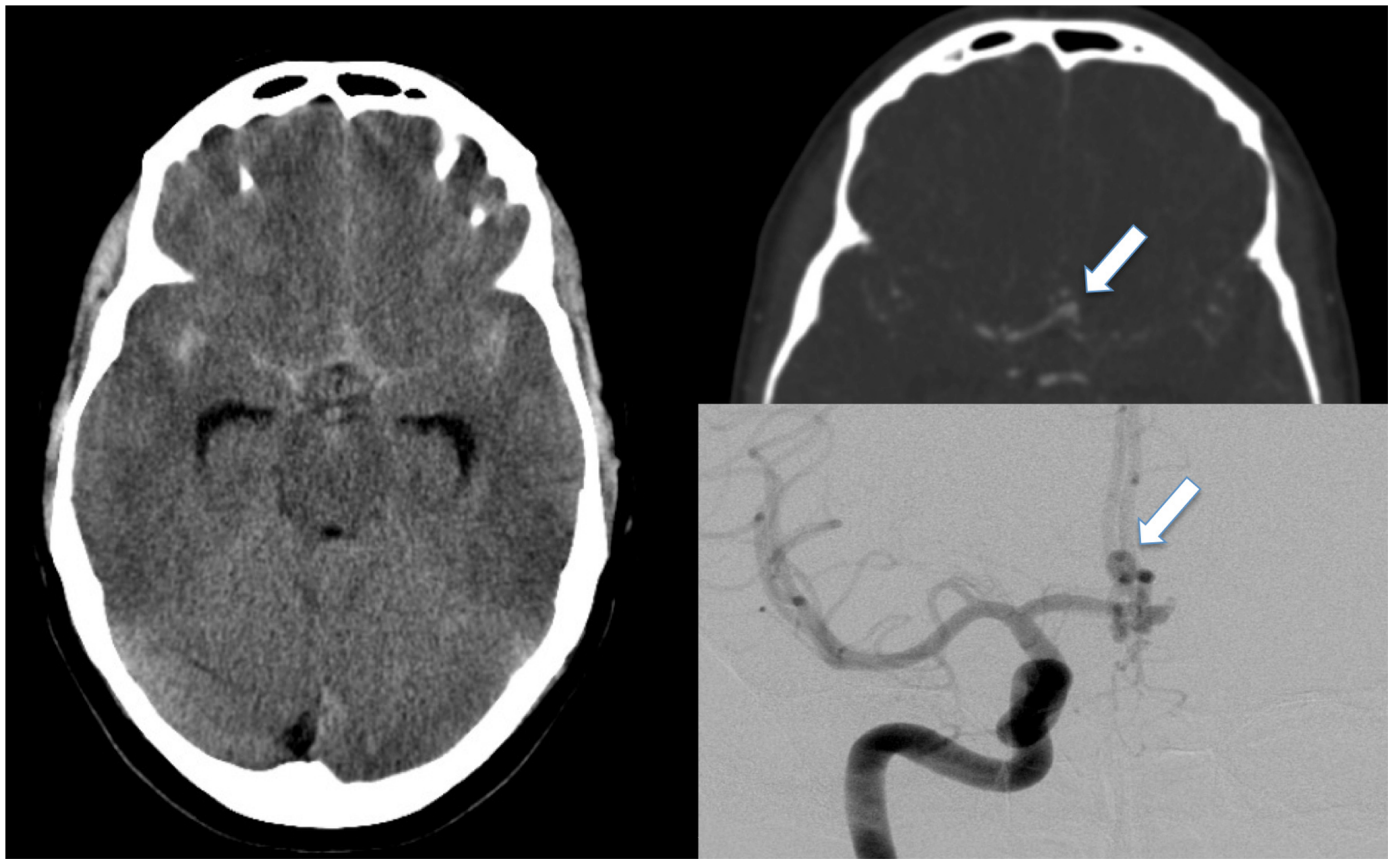
45 year old woman presenting with acute thunderclap headache. Inital CT showed subarachnoid hemorrhage. Contrast enhanced CTA revealed an arteria cerebri anterior aneurysm with a diameter of 3(DSA).

Eighty point seven percent (n = 42) of the radiological findings were a sexual intercourse-associated pathology and 19.2% (n = 10) were considered to be incidental findings. Pathological findings were neither associated with gender (p<0.50) nor age (p<0.78). For an overview on radiological findings see table 3. Radiological images of pathologies associated with sexual intercourse related emergency department admissions can be found in figures 1, 2, 3, and 4.

**Table 3:** Radiological findings (n = 52).

	number (n)	percent (%)	
**sexual intercourse-associated radiological finding**	42	80.7	
cerebrovascular insults	21	40.4	
epididymitis	7	16.7	
obstructive uropathy	5	12.0	
ruptured ovarian cyst	2	3.9	
urolithiasis	1	1.1	
ocular bulbus perforation	1	1.1	*see * *figure 1*
foreign body inclusion	1	1.1	
penile hematoma	1	1.1	
hip luxation	1	1.1	*see * *figure 2*
type B aortic dissection	1	1.1	*see * *figure 3*
testicular abscess	1	1.1	
**incidental findings**	10	19.3	

Of the patients with cerebrovascular insults 71.4% (n = 15) were male and 28.6% (n = 6) female. The median age was 49 years (SD 12.55, range 22–67). In contrast to other patients with positive radiologic findings cerebrovascular insult was found significantly more often in men (p<0.0001). Patients with cerebrovascular insults were significantly older than those with other positive radiological findings (p<0.0001). The most common pathology was atraumatic subarachnoid hemorrhage (n = 14, 66.6%), followed by atraumatic intracerebral hemorrhage (n = 4, 19.0%), two ischemic cerebrovascular insults and one atraumatic subdural hemorrhage. The most common grade of subarachnoid hemorrhage was Fisher grade two (n = 8, 66.6%), followed by two cases of grade one and each one case of grade 3 and 4. Of the patients with atraumatic subarachnoid hemorrhage two cases of ruptured cerebral artery aneurysm were detected by digital substraction angiography (see figure 4).

## Discussion

In our emergency department complications of sexual intercourse leading to immediate emergency department admissions are rare and account for only 0.1% of all patients admitted to our emergency department annually.

In our study the most common pathological finding was headache attributed to non-traumatic hemorrhage, most often subarachnoid. It has been reported that 14.5% of all non-traumatic subarachnoid hemorrhages are precipitated by sexual activity [4], [9]. Intracranial aneurysm ruptures leading to subarachnoid hemorrhage occur in 3.8%–4.5% [5]. According to a study by Fann et al the risk of atraumatic subarachnoid hemorrhage is increased 15-fold as the acute elevation in blood pressure during sexual intercourse increases the vessels wall tension and the subsequent risk of its rupture [10]. Similar as in our study several other studies found a male predominance in patients with atraumatic subarachnoid hemorrhage related to sexual intercourse [4], [5], [11], [12]. This finding is exceptional for several reasons [5]. As women may experience multiple orgasms and the duration of their orgasm is longer than those of men one would expect women to be more susceptible to subarachnoid hemorrhage related to sexual intercourse [5]. Furthermore the overall incidence of subarachnoid hemorrhage due to aneurysms is higher in women than in men [13]. In our study cerebral computer tomography was used far more often to image subarachnoid hemorrhage than MR. According to a review by Brazelli et al CT and MRI are equal in detecting acute vascular lesions in patients presenting with stroke symptoms [14]. Computer tomography has some advantages such as being less costly, quicker to perform and easier to tolerate [14]. Nonetheless subarachnoid hemorrhage may not be detected on CT either because there is only a small amount of subarachnoid blood present or because too much time has elapsed between the time of hemorrhage and the time of imaging [15], [16]. Several studies suggest that MRI is as sensitive as or more sensitive than CT scanning in the evaluation of acute subarachnoid hemorrhage, however, compared with lumbar puncture neither MRI nor CT can exclude SAH [17].

More than 50 percent (38/55) of the cerebral imaging performed in our study did not show any pathological findings. In these cases post-coital headache and transient global amnesia were diagnosed. Both are common in patients presenting themselves with neurological symptoms after sexual intercourse [3], [18]. According to a study by Kirz et al. on coitus as a predisposing factor for neurological symptoms about 50% of all patients suffer from post-coital headache [19]. Transient global amnesia is estimated to be related to sexual activity in up to 5% of cases as described in a review by Larner et al [20].

According to the IHS classification (International Headache Society) headaches that occur in relation to sexual intercourse are of explosive character (similar to thunderclap headache) which lead to a most intense headache within less than one minute [21]. The abrupt onset and the acute character mimics subarachnoid hemorrhage or cerebral ischemia [20], [22], [23]. Neither post-coital headaches nor amnesias can be identified with a radiological examination, but cerebral imaging and lumbal puncture should be performed to rule out potentially dangerous differential diagnoses [20], [22]–[24].

Ischemic cerebrovascular insult was rare in our study as well as in the literature. Only case reports on this topic exists [25], [26]. Possible pathophysiologies are cerebral vasospasm or paradoxical embolization in patients with a patent foramen ovale [27].

Patients with admission due to infectious causes in relation to sexual intercourse were the second most common reason for radiological imaging in our study. The association between urinary tract infections and sexual intercourse is generally well known [28]. Several studies regarding risk factors for lower and upper urinary tract infections showed that incidences are increased with the frequency of sexual intercourse, numbers of partners, the change of partner, oral or anal intercourse as well as the use of lubricants and spermizids [28], [29]. Especially in women the mechanical action of sexual intercourse facilitates entry of *E. coli* strains into the bladder, and both sexual intercourse and spermicide use alter the normal lactobacillus-dominant vaginal flora and facilitate *E. coli* colonization of the vagina [29].

Some of our patients with urinary tract infections showed signs of obstructive uropathy with urinary tract infects. The majority of the majority of urinary tract infection are often uncomplicated and do not require emergency radiological investigation [30]. This explains why in our study only 21 out of 97 patients with urinary tract infections had an urogenital sonography. The main aim of urogenital sonography is to rule out complications such as obstruction or abscess [30], [31]. It is generally recommended to be performed within the first 24 hours [30] after inicial presentation. It is possible that some urogential sonographies were performed in a stationary setting outside of the ED and therefore some pathological findings were not observed.

As in our study urolithiasis may be precipitated by sexual intercourse. According to Wilson et al the adrenergic stimulation of the ureteric smooth muscle as well as the changes in body-position are responsible for urolithiasis becoming symptomatic during sexual intercourse [32].

Aortic dissection related to sexual intercourse has so far only been described in a single case report [33]. The risk of aortic dissection is estimated to be increased during sexual intercourse due to the increased blood pressure and concomitant aortic wall tension [33]. But due to the small number of reports concerning aortic dissection in relation to sexual intercourse an incidental finding rather than true coherence cannot be excluded.

### Limitations

One major limitation of this study is that it covers only sexual activity related emergency department admission of patients that stated that the reason for presentation was attributed to prior intercourse. Our data therefore almost certainly underreport the actual prevalence of sexual activity related emergencies. All patients that presented with a delay of some days with sexual activity related emergencies, such as signs of abdominal pain in terms of pelvic inflammatory disease, were only included in this study if they stated that their problems started in direct relation to sexual intercourse (within 24 hours). All other patients were not included in this study, a fact that leaves room for bias and lowers the generalizability of our work. For more detailed data as well as for patients with delayed conditions attributed to sexual intercourse and their radiological work-up needs to be assessed in a prospective study. An additional limitation is that as sexual health in Switzerland is managed by family physicians, gynaecologists and specialized outpatient clinics it is likely that some patients were referred to these services during office hours. The data were collected retrospectively from narrative comments in notes which means that information bias and interpretation bias cannot be excluded. We did not assess chronic medical conditions and we therefore do not know how many of our patients were suffering from underlying diseases such as hypertension. Additionally as the treatment of patients with sexual intercourse related emergency department admissions is not standardized at our hospital our results have low external validity and depend highly on our hospital. Furthermore because patients younger than 16 years and gynaecological emergencies are treated in different emergency departments in our hospital, underreporting of emergencies characteristic for women or adolescents younger than 16 years are possible.

### Conclusion

Radiological examinations are often performed in the setting of immediate emergency department admissions in relation to sexual intercourse. Pathological findings are manifold. Cerebral imaging is the most common type of radiological imaging performed.

As this study only presents a first overview on radiological imaging in patients with sexual intercourse related emergency department admissions further prospective and standardized studies should be performed to better evaluate the significance of radiological imaging in this patient collective with the aim to gain better knowledge on what patients profit from what type of radiological imaging when presenting with a sexual intercourse related emergency.
